# Investigating materials and orientation parameters for the creation of a 3D musculoskeletal interface co-culture model

**DOI:** 10.1093/rb/rbaa018

**Published:** 2020-05-01

**Authors:** Hamad Alsaykhan, Jennifer Z Paxton

**Affiliations:** r1 Anatomy@Edinburgh, Edinburgh Medical School: Biomedical Sciences, The University of Edinburgh, Teviot Place, Edinburgh EH8 9AG, UK; r2 Department of Basic Medical Sciences, College of Medicine and Health Sciences, Qassim University, PO Box 991, 51911 Unaizah Campus, Al-Qassim 51911, Saudi Arabia

**Keywords:** tendon, bone, 3D, co-culture, hydrogels, musculoskeletal, interface

## Abstract

Musculoskeletal tissue interfaces are a common site of injury in the young, active populations. In particular, the interface between the musculoskeletal tissues of tendon and bone is often injured and to date, no single treatment has been able to restore the form and function of damaged tissue at the bone–tendon interface. Tissue engineering and regeneration hold great promise for the manufacture of bespoke *in vitro* models or implants to be used to advance repair and so this study investigated the material, orientation and culture choices for manufacturing a reproducible 3D model of a musculoskeletal interface between tendon and bone cell populations. Such models are essential for future studies focussing on the regeneration of musculoskeletal interfaces *in vitro*. Cell-encapsulated fibrin hydrogels, arranged in a horizontal orientation though a simple moulding procedure, were shown to best support cellular growth and migration of cells to form an *in vitro* tendon–bone interface. This study highlights the importance of acknowledging the material and technical challenges in establishing co-cultures and suggests a reproducible methodology to form 3D co-cultures between tendon and bone, or other musculoskeletal cell types, *in vitro.*

## Introduction

Tissue engineering is defined as the development of tissues or organs by manipulating biological, biophysical and biochemical factors in a laboratory setting [1]. Engineering a 3D model of tissues is a technique used in many laboratories worldwide, yet few have used this approach to model a musculoskeletal interface such as the enthesis. The enthesis is the biological and mechanical junction between tendon and bone [2, 3]. It is commonly injured in young, active populations, e.g. such as anterior cruciate ligament injuries [4] tennis elbow, jumper’s knee, rotator cuff tendon tears and calcaneal tendon avulsion [2, 3, 5]. Also, the enthesis is vulnerable to injury via enthesopathy diseases, spondyloarthropathy diseases, falls and automobile accidents [6, 7] as well as degeneration through normal ageing, particularly in the rotator cuff tendon group [8]. Importantly, the native enthesis possesses a unique microanatomical transition between the soft and hard tissues that fails to be replicated following injury to this region [3, 9]. Instead of a gradual transition between the hard and soft tissues that acts as a suitable structure for force transfer, the injured enthesis is composed mainly of a weakened scar tissue that remains susceptible to further injury [2, 3]. As such, research into methods to help restore the natural gradation of the enthesis and its mechanical function following injury is much needed.

To investigate and understand the important events occurring at the enthesis during formation or injury and repair, an *in vitro* model would be an invaluable research tool. Indeed, a previous study has highlighted important osteoblast-fibroblast interactions in standard 2D cell culture model [10] but this has yet to be replicated in a 3D environment. It is now well documented that traditional 2D cell culture methods do not represent the native tissue environment and that many cellular characteristics are altered when comparing 2D to the 3D counterparts [11]. Therefore, the main focus of this study was to establish the formation of a 3D co-culture *in vitro* model to enable future investigations into the enthesis and bone–tendon 3D co-cultures to be undertaken.

Scaffolds are the basis of most 3D tissue-engineered products. A scaffold in 3D tissue engineering acts as an artificial extracellular matrix (ECM) to mimic the biological and mechanical properties of native tissue [12]. The natural ECM provides the tissue with structural integrity and mechanical properties like stretching, resistance and weight bearing. It is also the ECM that stores different growth factors and facilitates their actions on cells [13]. Choosing a scaffold for design of a tissue-engineered product involves consideration of many requirements including architectural design, material biocompatibility, biodegradability and manufacturing technologies. In addition, there are many potential scaffold candidates available, each with their own advantages and disadvantages [14, 15].

In this study, four commonly used scaffold materials in the field of tissue engineering were investigated to form a co-culture between two distinct cell type populations in 3D; (i) agarose [16, 17], (ii) gellan [18–20], (iii) fibrin [21–23] and (iv) collagen [16, 24–26]. A system was designed to host two cell-encapsulated hydrogels in a co-culture, in either a vertical or horizontal arrangement. Hydrogels were considered as suitable candidates due to their superior flexibility to form shapes of their surrounding mould or container and their ability to allow homogenous cell distribution throughout the cell-encapsulated hydrogel. As the scaffold needed for cell-encapsulated co-culture experiments was intended to be replaced by ECM formed by the cells, natural biodegradable hydrogels were assessed. The candidate hydrogel to be used for cell-encapsulation co-culture and ECM assessment had to meet specific criteria, including the hydrogel being of adequate form to allow co-culture formation with a single interfacial boundary between cell types, allow cells to attach, support cell proliferation, not cause significant cell death during the preparation and cell encapsulation processes and show consistent and reproducible results. We predict that success in forming a 3D co-culture *in vitro* model will be a valuable research tool for notable enthesis investigations of the future.

## Materials and methods

### Hydrogel materials

#### Agarose

Agarose hydrogels were prepared by mixing 1 g of UltraPure™ low melting point agarose powder (Invitrogen, UK) with 99 ml of distilled water and temperature was raised gradually until the powder fully dissolved to a final concentration of 1% agarose solution. The agarose was sterilized by autoclaving. Cell solution was mixed with agarose at no more than 40°C inside a laminar flow hood in a 1:1 ratio to result in 0.5% agarose hydrogel with suspended cells. The 0.5% cell-suspended agarose was freshly prepared for each experiment and cultured at 37°C, 5% CO_2_ for the duration of each experiment.

#### Gellan

Gellan powder was hydrated by mixing with deionized water at 70–80°C temperature. After complete hydration of the powder, the gellan hydrogel was autoclaved immediately. The sterile gellan hydrogel was transferred to a laminar flow hood to be mixed with cells in a 1:1 ratio at a temperature not higher than 40°C.

#### Fibrin

Preparation of fibrin hydrogel used sterile solutions of fibrinogen (20 mg/ml) and thrombin (200 U/ml). Thrombin mix was prepared by adding 97.1% cell suspension in supplemented DMEM [Dulbecco’s modified Eagles medium (sDMEM)] including 10% foetal bovine serum (Labtech, UK), 2.4% l-glutamine (Life Technologies, UK), 2.5% 4-(hydroxyethyl)-1-piperazineethanesulphonic acid buffer (Life Technologies, UK) and 1% penicillin/streptomycin (Life Technologies, UK), 2.4% thrombin, 0.2% aprotinin and 0.2% aminohexanoic acid. To make fibrin hydrogel, a solution of one-part fibrinogen and five parts thrombin mix with encapsulated cells was made. The construct was then incubated for 1 h to allow the hydrogel to polymerize.

#### Collagen

Mixing nine parts of collagen hydrogel (pH 2) with one part 0.2 sodium phosphate (pH 11.2) resulted in an optimum collagen hydrogel for 3D cell encapsulation (pH 7), which had a final concentration dilution of 6 mg/ml pepsin soluble collagen as supplied and described by the manufacturer (Collagen Solutions, UK). sDMEM was used to dilute collagen to a final concentration of 3 mg/ml. Collagen was kept at a temperature of 2–10°C for storage and during cell encapsulation. Polymerizing collagen hydrogel was achieved by incubation it at 37°C, 5% CO_2_.

### Cell culture

#### Cell sources

##### Chick tendon fibroblasts with or without green fluorescent protein label

Embryonic chick tendon fibroblasts with or without green fluorescent protein label (CTF/CTF-GFP) were isolated from metatarsals tendons of dissected hind limbs of chick embryos/GFP-tagged chick embryos on Day 13.5. Dissected tendons were placed in 5% antibiotic/antimycotic (ABAM, HyClone, GE Healthcare Life Sciences) in phosphate-buffered saline (PBS) solution. After three washes with sterile PBS in the laminar flow hood, the cells were isolated from tendon by submerging in 0.1% collagenase type II DMEM and incubated for 1.5 h at 37°C, 5% CO_2_. The cells were isolated from the solution by using a 100-µm cell strainer (BD Falcon, USA). Cells were moved to a T-175 cm^2^ flask and incubated at 37°C, 5% CO_2_ and cultured according to a general culturing procedure. CTFs with GFP tag were used for visualization and tracking studies and non-GFP-tagged CTF cells were used for viability studies.

##### Mouse osteoblasts (MC3T3)

This cell line from mouse calvaria was acquired from the European Collection of Authenticated Cell Cultures (ECACC, UK). These cells are osteogenic precursor cells that can differentiate into osteoblasts and osteocytes. They were thawed upon receiving, cultured in sDMEM and incubated at 37°C, 5% CO_2_ for the duration of all experiment*s*.

###### Cell labelling

Red cell tracker was used to label MC3T3 cells (CellTracker™ Red CMTPX, Life Technologies, UK) Cell tracker working solution was prepared according to the manufacturer’s protocol to a final concentration of 15 µM. Briefly, 50 µg of cell tracker powder were dissolved in 7.3 µl of DMSO to make a 10-mM cell tracker dye solution. This was followed by diluting the solution to a standard working concentration of 15 µM of cell tracker dye. CellTracker™ was used to label MC3T3 cells in all visualization and tracking experiments and non-labelled MC3T3 cells were used in the assessment of cellular viability experiments.

### Co-culture system development

#### Vertical vs horizontal orientation

##### Vertical orientation system development

**Figure 1:**
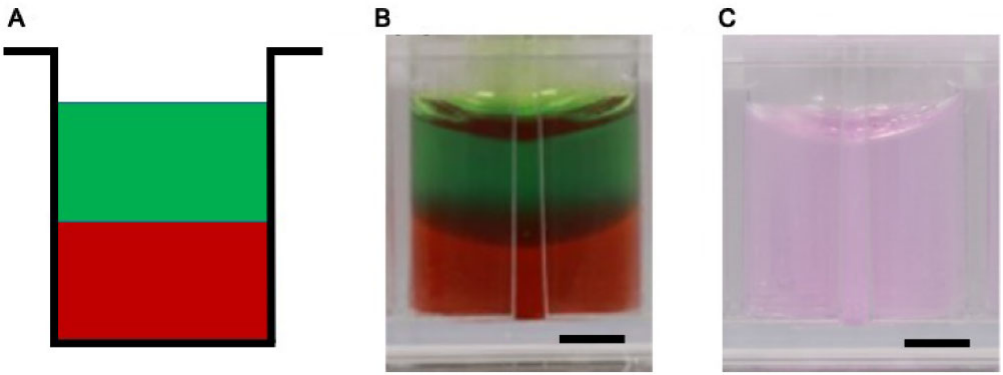
Design for making a 3D co-culture interface in the vertical orientation. (**A**) Schematic diagram displaying the layering design to make a 3D co-culture model. (**B**) Gross assessment of agarose hydrogel in a layered co-culture design pseudocoloured for clarity. Note the concavity of the gel layers at the interface. (**C**) Agarose co-culture manufactured without colour (scale bar = 2 mm).

To create a single interface between two stacked hydrogel layers, hydrogels of agarose, gellan, fibrin and collagen hydrogels in flat-bottomed, cell repellent 96-well plates (Greiner Bio-One, UK) (Fig. 1). Gross assessment was performed using red and green food colourings with hydrogels to help visualize the formation of the single interface (Fig. 1B).

###### Cell-free vertical interface assessment

For each hydrogel, 80 µl of two differently coloured layers were stacked in a single well (Fig. 1). A side-view image was taken at Day 0 for each stacked layers of hydrogels by a digital single-lens reflex camera (Canon D6 DSLR, Canon, Japan) equipped with a 100-mm macro lens (Canon EF 100 mm f2.8 USM Macro Lens, Canon, Japan).

###### Cell-encapsulated vertical interface assessment

Microscopic evaluation of the formed interface was implemented. In this methodology, CTF cells were encapsulated in a hydrogel (50 K cells/100 µl) and 80 µl was cast at the bottom of the well. Following setting of the hydrogel, 80 µl of a hydrogel encapsulated with MC3T3 cells were cast on the top. After the hydrogel set, sDMEM was added to each well and the construct was assessed by confocal laser scanning microscopy (CLSM) immediately after formation (at Day 0). Datasets of images were analysed and processed using Imaris software (Bitplane, Oxford Instruments, UK).

##### Horizontal orientation system development

###### Half-well silicone moulds

**Figure 2:**
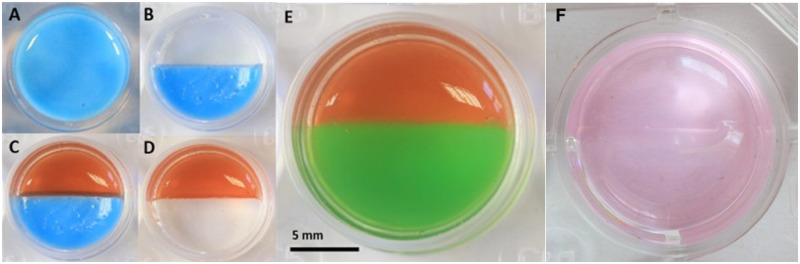
Bespoke well design for making a 3D co-culture interface in the horizontal orientation. (**A**) Silcone gel was set inside the well of a 24-well plate. (**B**) A half-well plug of silicone was inserted in the well. (**C**) A cell-encapsulated hydrogel poured in the exposed side of the system. (**D**) After the hydrogel solidifies, the silicone is removed exposing the other side of the system. (**E**) The other cell type is encapsulated in the hydrogel and poured in the empty space (pseudo red and green colours were used with agarose gel for demonstration purposes only). (**F**) Actual 3D co-culture produced with agarose hydrogel.

Half-well moulds were created to seal one side of a tissue-culture well while a cell-encapsulated scaffold was formed (Fig. 2). These half-well moulds were made by pouring Kemsil silicone (Kemdent, UK) prepared according to the manufacturer’s instructions in a 24-well plate wells (Greiner Bio-one, UK). The Kemsil silicone polymerized in 10 min creating a well-plug which was collected from wells and cut in half using a scalpel (Swann-Morton, UK). The half-well plugs were sterilized by submerging in 70% alcohol for 30 min and placed in a laminar flow hood for 30 min to dry before use.

##### Implementation of horizontal orientation system design

A simple, yet novel, system was designed to allow the production of two cell-encapsulated hydrogels in a side-by-side orientation to form a musculoskeletal interface model with one single 3D interface between hydrogels. To accomplish this, half-well silicone moulds were made (Fig. 2A and B) and were placed into an empty well to seal one side of a 24-well culture well (Greiner bio-one, UK) (Fig. 2B). A single cell hydrogel (300 µl) was cast into the exposed area of the well (Fig. 2C). Once set, the silicone mould was removed (Fig. 2D) and the other hydrogel was pipetted into the empty portion of the well (Fig. 2E), creating a 3D interface model between two cell-encapsulated hydrogels.

##### Cell-free horizontal interface assessment

Two volumes of agarose, gellan, fibrin and collagen hydrogels were coloured separately as red and green. The two coloured hydrogels were used in the system as described in Section 3.3.1.3 to create a single interface. Top-view images were taken at Day 0 for the formed interfaces by a digital single-lens reflex camera (Canon D6 DSLR, Canon, Japan) equipped with a 100-mm macro lens (Canon EF 100 mm f2.8 USM Macro Lens, Canon, Japan). The same datasets of images were used to assess the side profile of the interface by using Imaris software (Bitplane, Oxford Instruments, UK).

##### Cell-encapsulated horizontal interface assessment

A cell concentration of 50 K/100 µl of hydrogel solution was used for all hydrogel types. A single 3D interface between two cell-encapsulated hydrogels placed beside each other was created using the method described in Section 3.3.1.3. The interface between the cell-encapsulated hydrogels was imaged by CLSM at the same location for all samples on Days 0, 1, 2 and 3.

## Cell viability

Live/dead staining was conducted using Calcein AM and propidium iodide (PI) staining. The dye solution was freshly prepared for each time-point of an experiment in a dark environment. The required amount of dye solution was prepared with sDMEM, supplemented with 0.7% of 50 µg/ml Calcein AM (Invitrogen Molecular probes^®^, UK), which stains live cells green, and 2% of 1 mg/ml PI (Sigma, UK) to stain dead cells red. Samples were incubated at 37°C, 5% CO_2_ for 1 h before being visualized by CLSM. Hoechst 33342 stain was also used to stain the nuclei of live cells. The stain is cell permeable, which allows it to bind to the cell DNA and emit fluorescence when exited at 360 nm. Emission was detected at 460 nm confocal microscope filter.

## Cell visualization in hydrogels

### Confocal laser scanning microscopy

Data sets of confocal images were obtained from an inverted confocal laser scanning microscope system (Nikon A1R, Nikon, UK). The system allowed for live imaging with culture plates unopened to maintain sterility and to permit visualization of the same well over multiple time points. Atmospheric lenses used were 4× and 10× according to the experimental needs. Laser intensity and detector gain were adjusted according to experimental needs considering labelling quality, number of cells, photobleaching, depth of images and background noise. Data sets obtained were analysed by NIS Elements (Nikon, UK), ImageJ (National Institute of Health, USA) and Imaris Software (Bitplane, Oxford Instruments, UK).

## DNA quantification

The CTF and MC3T3 cells DNA content of the cell-encapsulated agarose and fibrin hydrogels were assessed (CyQuant™ cell proliferation assay, Invitrogen, UK). On the day of cell-encapsulation, four samples of each cell/hydrogel combination were stored in a −80°C freezer as Day 0 samples (*N* = 3, *n* = 4). Other four samples were cultured for 7 days then washed with 1× PBS and stored in the −80°C freezer (*N* = 3, *n* = 4). The CyQuant™ cell proliferation assay was not designed to assess DNA content of cells encapsulated in hydrogels. Therefore, hydrogel-specific cell-isolation protocols were used before starting the CyQuant™ cell proliferation assay. On the day of assay, all samples were thawed, and hydrogel-specific preparation was performed as described below:

### Agarose hydrogel

The cells encapsulated in agarose were retrieved by incubating samples at 75°C for 30 min and centrifuging the cells for 5 min at 2000 rpm (1-13 microfuge, Sigma, UK). Agarose was discarded, and the pellet of cells was used for the CyQuant™ cell proliferation assay as described by manufacturer to quantify DNA content.

### Fibrin hydrogel

#### Homogenization of fibrin hydrogel method for DNA measurements

Retrieval of DNA of CTF and MC3T3 cells-encapsulated in fibrin hydrogel was attempted. The cell-encapsulated fibrin hydrogel was minced with a disposable sterile scalpel blade number 10a (Swann-Morton, UK). The minced cell-encapsulated hydrogel was sonically disrupted to form a homogenized solution (SSE-1 Digital Sonifier, Branson, UK). The homogenized solution was used for the CyQuant™ cell proliferation assay as described by the manufacturer to quantify DNA content.

#### Use of nattokinase for fibrin hydrogel digestion and cell retrieval

As described in [27], the use of nattokinase enzyme fibrinolytic activity to retrieve cells encapsulated in fibrin hydrogel was performed with a small modification to digestion time. Nattokinase powder (NSK-SD; Japan Bio Science Laboratory Co. Ltd., USA) was dissolved in PBS containing 1 mM EDTA to a final concentration of 50 FU/ml (Sigma, UK). Samples in 1.5 ml microtubes were washed with 1× PBS then, 1 ml of nattokinase solution was added to samples and cultured at 37°C, 5% CO^2^ overnight (in the Carrion *et al.* study, the digestion period with nattokinase was from 30 to 90 min using fibrin hydrogels of lower concentrations). After incubation, samples were centrifuged at 1000 rpm for 3 min then used for CyQuant™ cell proliferation assay.

## Statistical analysis

Excel software was used on all quantitative data to calculate averages and determine standard curves (Excel 2016, Microsoft Office, USA). Statistical tests were performed using GraphPad Prism (Version 8.1.1, GraphPad Software Incorporation, USA). Analysis was performed using paired *t*-test to compare Days 0–7 viability results. Data presented as ‘*Nn*’ where ‘*N*’ represents number of independent experimental repeats, whereas ‘*n*’ represents number of technical replicates per experiment.

## Results

### Comparing vertical and horizontal 3D co-culture models to represent a musculoskeletal tissue interface

3D co-culture models were assessed through gross morphology, co-culture feasibility and ease of preparation to compare vertical (Fig. 1) to horizontal (Fig. 2) orientations. In the vertical arrangement, all gel types permitted the formation of two separate hydrogels touching at one single interface plane (Fig. 1). While discerning individual hydrogel layers was not easy in the standard hydrogels (Fig. 1C), using pseudocoloured hydrogels with food colouring permitted the visualization of the two individual hydrogel layers (Fig. 1B). Notably, the hydrogel layers appear concave due to surface tension of liquid on the wells of the culture plate (Fig. 1B).

Similarly, in the horizontal arrangement, all gel types permitted the formation of two separate hydrogels in contact at one single interface, as shown in agarose gel with the addition of pseduocolours (Fig. 2E) and as prepared normally (Fig. 2F).

**Figure 3:**
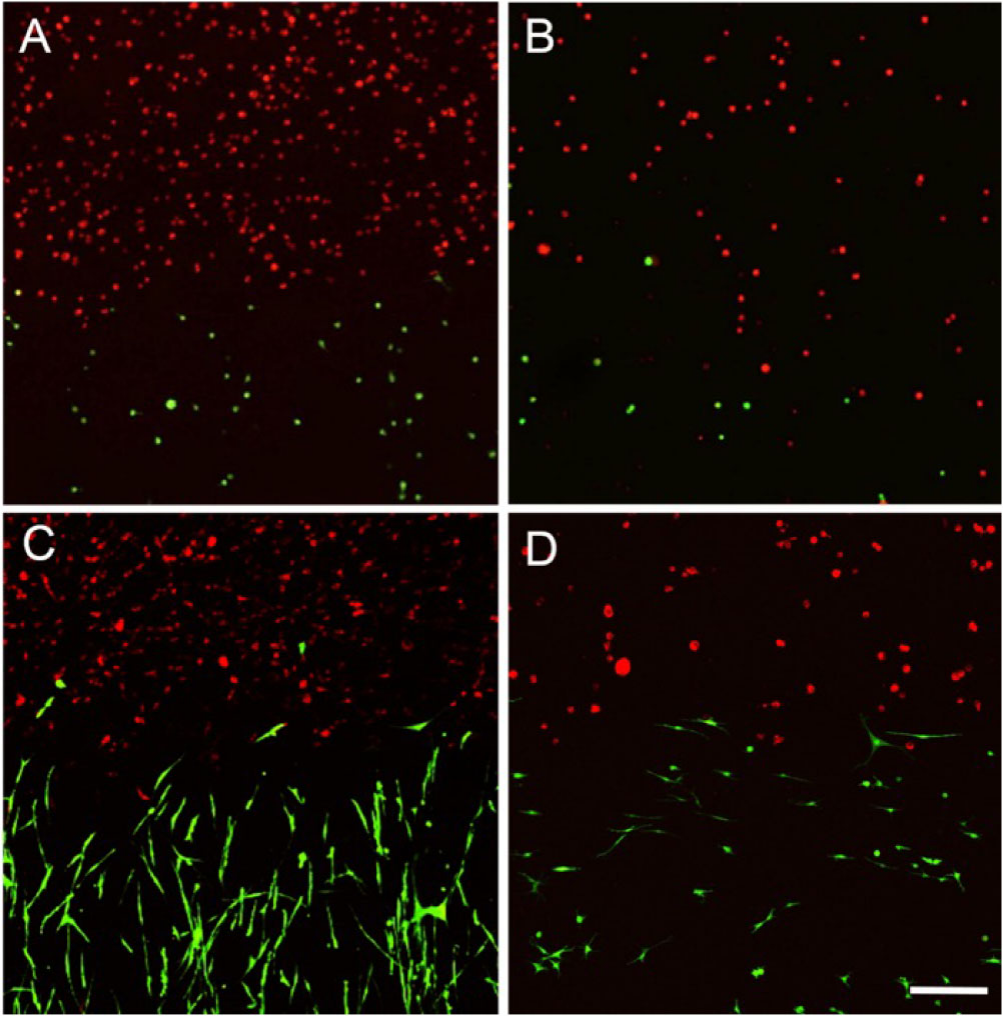
Horizontal system assessment. CLSM datasets were acquired at Day 1 and processed using ImageJ to stack a *Z*-axis projection of the total signal in the dataset. Green-labelled cells were CTF, whereas red-labelled cells were MC3T3 cells in (**A**) agarose, (**B**) gellan, (**C**) fibrin and (**D**) collagen hydrogels. The CTF and MC3T3 cells occupied opposite sides of the field and are in direct physical contact (scale bar = 200 µm).

**Figure 4:**
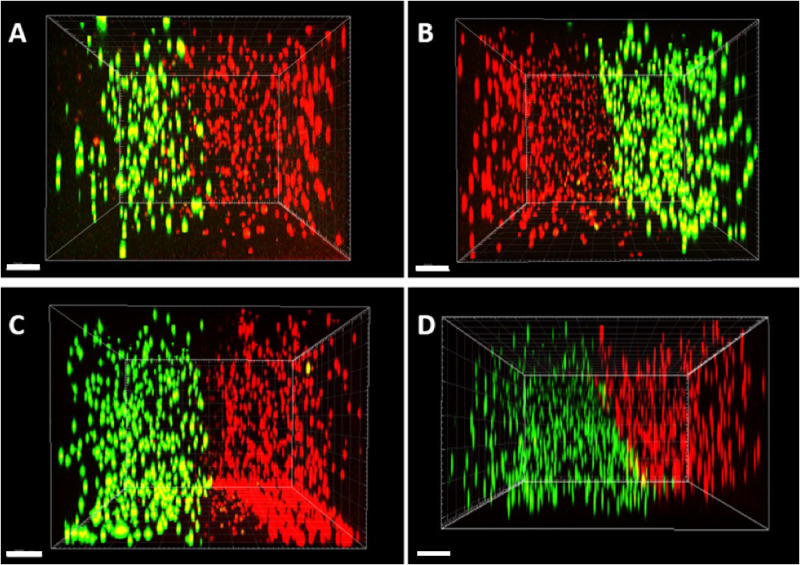
Assessment of the interface plane by examining the side profile of the dataset obtained by CLSM at Day 0 in the horizontally orientated system. (**A**) Agarose, (**B**) gellan and (**C**) fibrin are showing an acceptably perpendicular interface and (**D**) collagen showed an angled interface (scale bar = 200 µm).

**Figure 5:**
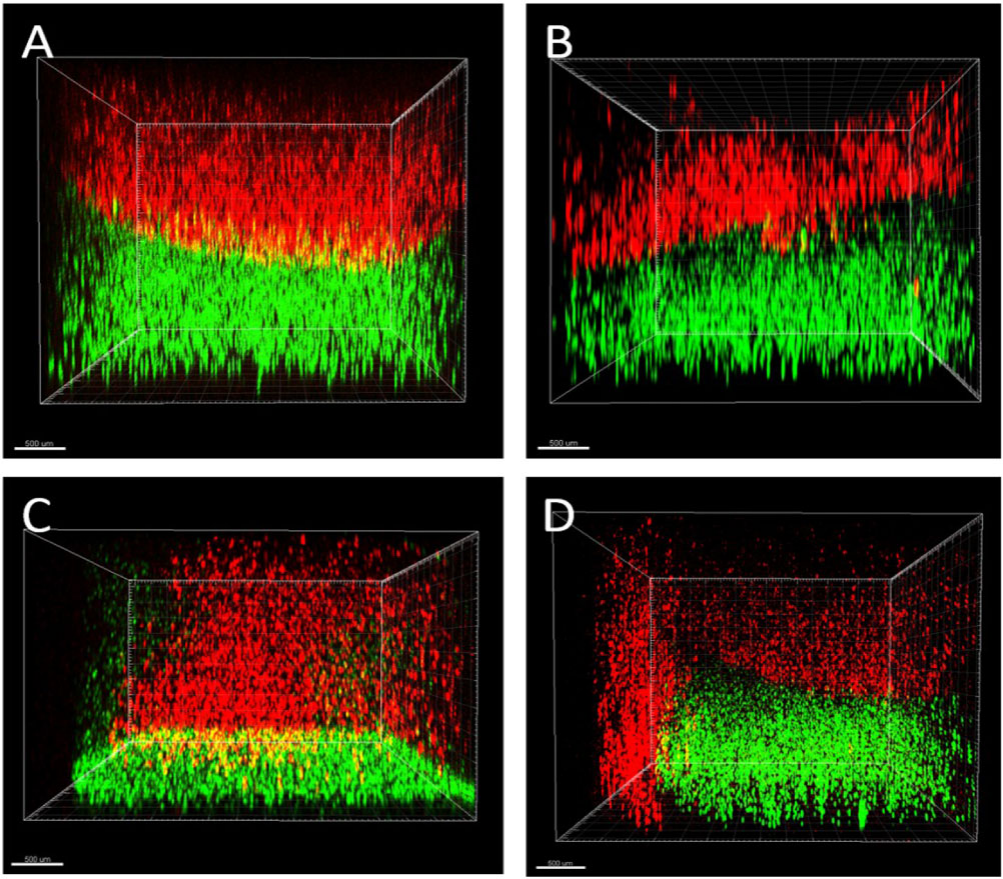
Vertical system orientation. CLSM was used to observe the formed vertical interface between MC3T3 (red) and CTF (green) cells at Day 0. (**A**) Agarose, (**B**) gellan, (**C**) fibrin and (**D**) collagen hydrogels were used to encapsulate MC3T3 (red) and CTF (green) cells. Surface tension affected the shape of hydrogels. More importantly, fibrin and collagen hydrogels showed signs of leakage as MC3T3 (red) cells were observed occupying parts of the bottom half of the vertical co-culture system (scale bar = 500 µm).

Although gross assessment of 3D co-cultures in both the vertical and horizontal systems appeared to show a clear demarcation between two hydrogels, the position of cells was then assessed to ensure a clear boundary existed between cell types on formation of the co-culture (Figs 3–5). In horizontal co-cultures, a clear boundary was present between bone (red) and tendon (green) cells in all four hydrogel materials (Figs 3A–D and 4A–D). Furthermore, the two cell populations were in direct contact at a single, perpendicular interface. In contrast, the vertical co-cultures displayed a more random placement of cell populations (Fig. 5). While agarose and gellan hydrogels performed fairly well at maintaining a double-layered vertical arrangement of cell-encapsulated hydrogels (Fig. 5A and B), fibrin and collagen hydrogels performed poorly (Fig. 3C and D). In particular, noticeable leakage of both cell types to opposite sides of the well was noted in both collagen and fibrin hydrogels (Fig. 5C and B) which was not appropriate in our proposed model design. Similar results were obtained on multiple repeats of this experiment and due to these inconsistences in cell placement, as well as concerns about the concavity of the hydrogels and potential limitations nutrient transfer through a vertical co-culture layered design, the vertical design was disregarded and all future work conducted in the horizontal co-culture design orientation.

### Novel horizontal system permits formation of a reproducible musculoskeletal interface model

3D co-cultures of bone and tendon cells were successfully manufactured using the novel co-culture system set up (Figs 3 and 4). To investigate the effect of the hydrogel material used on the overall effectiveness of model creation, for different hydrogel materials were trialled in the system; agarose, collagen, gellan and fibrin (Fig. 5). On Day 0, all materials demonstrated a clear demarcation between the two cell types as expected based on previous findings (Fig. 5Ai, Bi, Ci and Di). In addition, cell morphology remained rounded, indicating a lack of cell attachment to the hydrogels at this early stage of the experiment (Fig. 5Ai, Bi, Ci and Di). By Day 1, cells remain rounded in agarose and gellan hydrogels (Figs 5Aii and 6Bii), but have transitioned to a more extended appearance, especially in the CTF cells, in fibrin and collagen hydrogels (Fig. 5Cii and 5Dii), indicating an attachment to the hydrogel substrate. Notably, CTF cells begin to invade the MC3T3 portion of the co-cultures in fibrin hydrogels from Day 1 onwards (Fig. 5Cii–iv), which is not evident in any other hydrogel material type.

### Importance of well plate treatment for maintaining 3D co-culture arrangement

**Figure 6:**
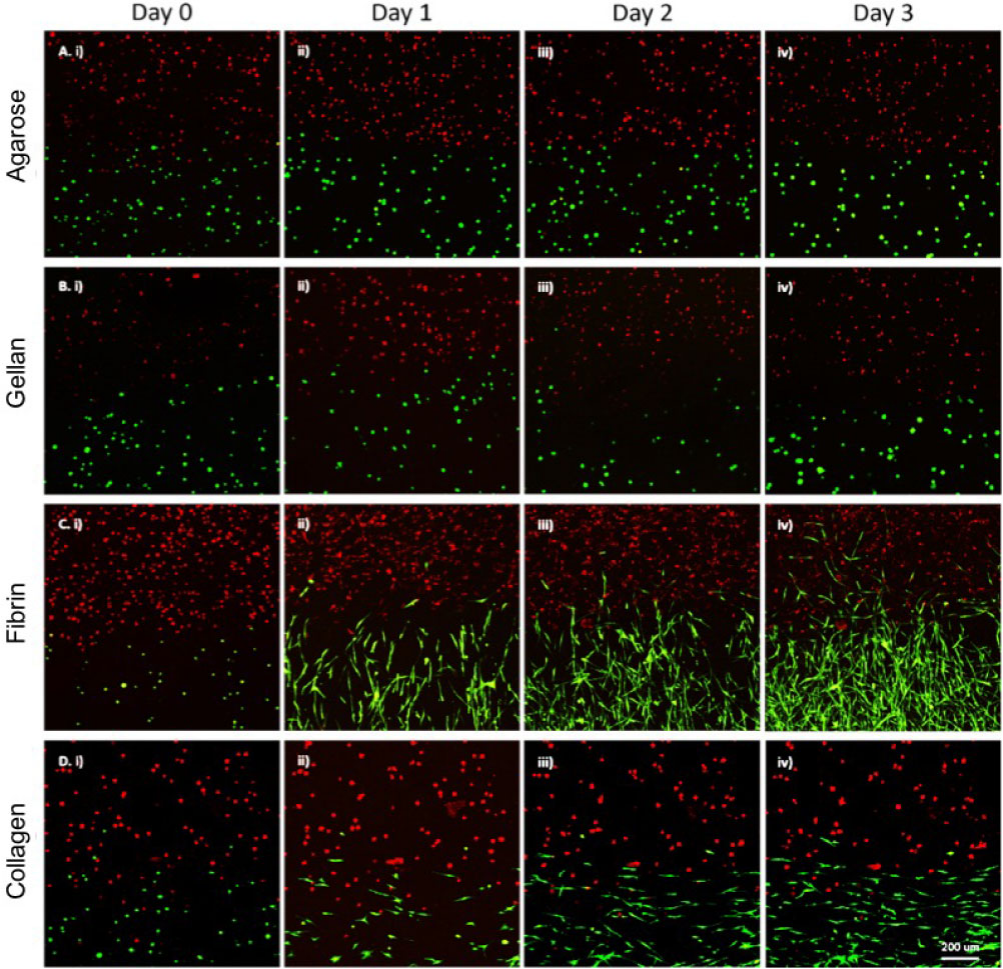
Short-term observation experiment to assess cell morphology in different hydrogels using CLSM. Noticeable differences in cell morphology could be observed comparing cells in agarose (**A**) and gellan (**B**) to cells in fibrin (**C**) and collagen (**D**). In (A) and (B) the cells were exhibiting a spherical morphology. In (C) and (D) the cells were exhibiting morphological changes relevant to their cell type. It is notable that CTF cells encapsulated in fibrin (C. iv) were showing migratory action by invading the MC3T3 side of the co-culture after 3 days of incubation. Note that tissue-culture treated 24-well plates were used.

**Figure 7:**
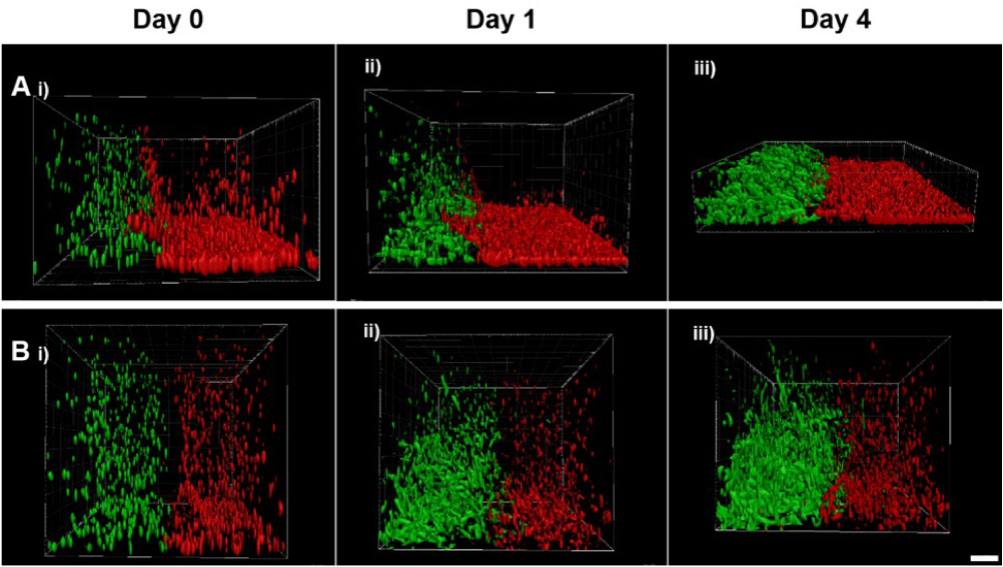
The importance of culture well treatment on cell placement by comparing side-plane projection view of the interface between two cell-encapsulated fibrin hydrogels. (**A**) Interface made using tissue culture treated 24-well plates showing presence of cells inside fibrin hydrogel at Day 0 followed by detection of cells at the plastic surface of the 24-well plate at Day 4, suggesting that cell crowdedness observed was due to proliferation on the plastic well surface. (**B**) Interface made using non-tissue culture treated 24-well plates showing a presence of CTF and MC3T3 cells inside the fibrin hydrogel throughout the experiment with an increase in cell crowdedness (scale bar = 200 µm).

While the images presented in Figs 4 and 6 demonstrated a clear interface produced between two cell-encapsulated hydrogels in the horizontal orientation, it was important to ascertain the side plane-projection distribution of cells to ensure that the encapsulated cells remained in 3D throughout the experimental procedure. Indeed, when cell-treated culture wells were used, both cell types quickly migrated through the gel to adhere to the plastic underneath, thus negating the 3D culture desired (Fig. 7Ai–iii). In contrast, forming 3D co-cultures in non-tissue treated wells maintained 3D cell distribution throughout the hydrogels (Fig. 7Bi–iii). This is an important, potentially overlooked, technical step to ensure that cells the 3D cultures are definitely retaining their 3D spatial orientation and are not accumulating on the bottom of tissue culture dishes.

### Assessing cell viability in hydrogels

Cell viability assessment in hydrogels can be achieved via several different methodologies and here, was an important factor for deciding a suitable hydrogel to be used for future studies on ECM formation and content. Here, the viability of cells encapsulated in both agarose and fibrin was assessed on Days 0 and 7 of culture by quantification of CLSM live/dead images to ascertain hydrogel suitability for longer-term cell culture using quantification of CLSM images.

**Figure 8:**
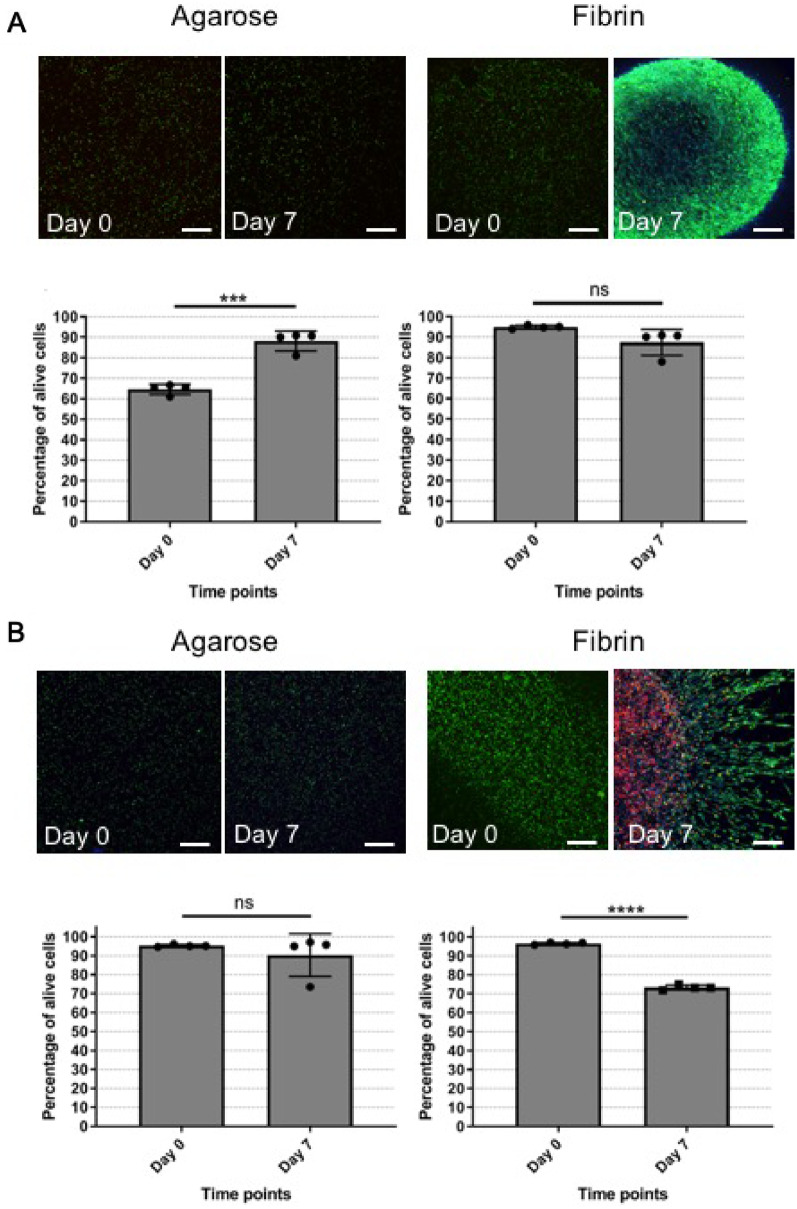
Hydrogel material influences cell viability. (**A**) CTF cells encapsulated in agarose and fibrin hydrogels. (**B**) MC3T3 cells encapsulated in agarose and fibrin hydrogels (paired *t*-test, *N* = 4, *n* = 4) (scale bar = 200 µm).

3D images of live/dead stains of cells encapsulated in agarose and fibrin hydrogels were statistically analysed (*N* = 4, *n* = 5; Fig. 8) The percentage of live CTF cells in agarose was significantly increased after 7 days of culture increasing from as 61.0% ± 2.5 on Day 0, compared to 88.2% ± 4.8 on Day 7 (Fig. 8A, B; *P* ≤ 0001). In contrast, the percentage of live cells was not significantly different between Day 0 (94.9% ± 0.8) and Day 7 (87.5% ± 6.3) when CTF cells were cultured in fibrin hydrogel (Fig. 8A and B; *P* > 0.05) indicating that fibrin hydrogels maintained cell viability well in the case of CTF cells.

For MC3T3 cells, encapsulation in agarose hydrogels lead to a non-significant difference in cell viability between Day 0 (95.3% ± 0.6) and Day 7 (90.3 ± 11.25) values (*P* = 0.4148; Fig. 8B). However, in fibrin hydrogels, MC3T3 cells decreased their viability from 96.4% ± 0.6 at Day 0–73.24% ± 1.3 at Day 7 (*P* < 0.0001; Fig. 8B).

Despite both hydrogel types maintaining reasonable levels of viability in both agarose and fibrin hydrogels, the live/dead assessment could not be used to confidently assess cell number, as viability is expressed as a percentage rather than absolute cell numbers to relate to proliferation, and so further investigations focussed on DNA content were undertaken.

### Cell number measured by DNA quantification

**Figure 9:**
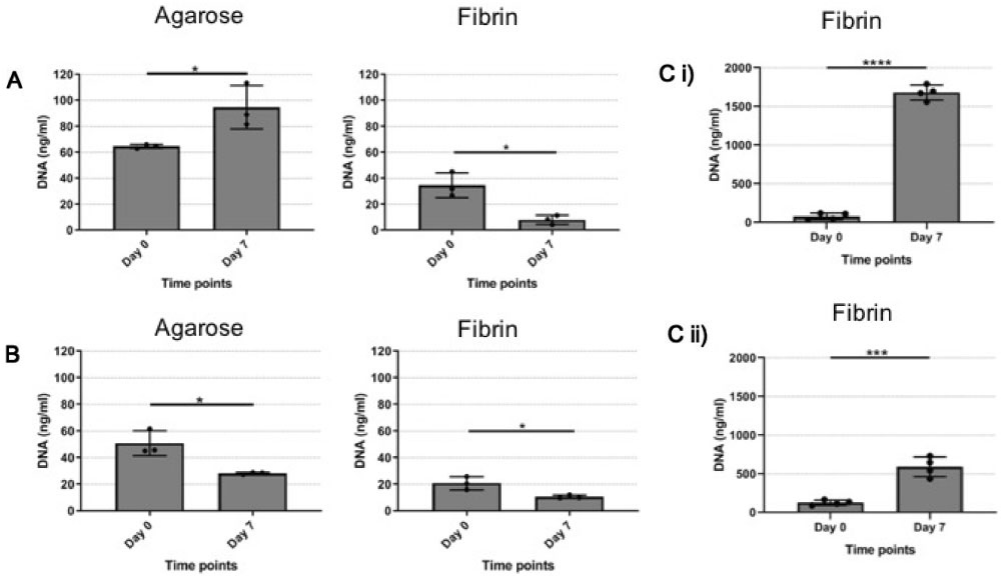
Method of cell retrieval for DNA analysis influences results. (**A**) CTF cells encapsulated in agarose and fibrin hydrogels (paired *t*-test, *N* = 3, *n* = 4). (**B**) MC3T3 cells encapsulated in agarose and fibrin hydrogels (paired *t*-test, *N* = 3, *n* = 4). (**C**) Fibrin hydrogels digested with nattokinase containing (i) CTF and (ii) MC3T3 cells (paired *t*-test, *N* = 4, *n* = 3).

Cellular proliferation was an important indicator for hydrogel suitability for future studies for longer-term co-culture work. Therefore, agarose and fibrin hydrogels support of cell proliferation was assessed by quantifying CTF and MC3T3 cells DNA content in the encapsulated hydrogels at Days 0 and 7 (Fig. 9).

#### Quantifying DNA of cells encapsulated in agarose hydrogel

CTF cells encapsulated-agarose DNA content had increased significantly from Day 0 (64.6 ng/ml ± 1.5) to Day 7 (94.7 ng/ml ± 16.6) (Fig. 9; *P* = 0.03). Similarly, MC3T3 cells DNA content was quantified when encapsulated in agarose hydrogel, but in this case showed a significant decrease from 50.9 ng/ml ± 9.4 at Day 0—28.3 ng/ml ± 0.8 at Day 7 (Fig. 9; *P* = 0.01).

#### Quantifying DNA of cells encapsulated in fibrin hydrogel

CTF cells DNA content fell sharply from Day 0 (34.62 ng/ml ± 9.52) to Day 7 (8.10 ng/ml ± 3.54) when encapsulated in fibrin hydrogel (Fig. 9A Fibrin; *P* = 0.01). Similarly, the DNA content of MC3T3 cells when encapsulated in fibrin hydrogel significantly decreased from 20 ng/ml ± 4.90 at Day 0–10.71 ng/ml ± 1.27 at Day 7 (Fig. 9B Fibrin; *P* = 0.03). This decrease of DNA content over time when cells were encapsulated in fibrin hydrogel was largely unexpected as qualitative data previously demonstrated a clear visual increase in CTF number qualitatively overtime (see Figs 6 and 8). It was therefore hypothesized that this could be due to using a poor cell retrieval methodology for fibrin hydrogels and so another methodology to recover cells from fibrin hydrogel was adopted from Ref. [27].

When using nattokinase in the cell retrieval step for fibrin hydrogels [27], cell numbers increased in line with the qualitative data collected previously (Figs 8 and 9). DNA quantification of CTF cells encapsulated in fibrin hydrogel showed an increase from 79.4 ng/ml ± 41.5 at Day 0—1680.0 ng/ml ± 95.9 at Day 7 (Fig. 9Ci; *P* ≤ 0.0001). Similarly, MC3T3 cells DNA quantification showed an increase from 128.0 ng/ml ± 32.3 at Day 0—591.7 ng/ml ± 127.0 at Day 7 (Fig. 9Cii); *P* = 0.0004. These results demonstrated that that cells encapsulated in fibrin hydrogel increased their cell density over time therefore strongly supporting the qualitative data presented earlier (Fig. 8).

In summary, these results strongly suggest that fibrin gel is the most suitable hydrogel for manufacturing 3D co-cultures to model musculoskeletal interfaces as they (i) form suitable, reproducible physical co-culture structures, (ii) support cellular attachment and proliferation and (iii) permit cellular migration between hydrogels to form an ‘interfacial region’. This simple hydrogel model can be used to assess cellular interaction and behaviour in future studies.

### Discussion

The main aims of this study were to design and develop a reproducible 3D co-culture system that could be used to model musculoskeletal interfaces *in vitro.* Through a series of physical and biological assessments, a simple-to-use system has been developed using inexpensive silicone moulding and cell-encapsulated fibrin hydrogels. This approach could be used to model musculoskeletal interfaces, such as the bone–tendon, bone–ligament, tendon–bone or cartilage–bone interfaces *in vitro.*

Although the original concept of the co-culture design appeared simple, our analysis of the physical difficulties in generating a reliable and reproducible model highlighted the difficulties that must be overcome, and potentially overlooked, when building *in vitro* models. For example, it could be assumed that the easiest, and most convenient method of building a co-culture model would be the vertical arrangement (Fig. 1) where one cell-encapsulated hydrogel was placed on top of another cell-encapsulated hydrogel. However, as we have shown in Fig. 3, problems with surface tension, and leakage of cells across the boundary at the time of manufacture (Fig. 5) limited the usability of this methodology. Furthermore, the curved upper surfaces proved to be problematic when imaging a distinct boundary at the interface between cell-encapsulated gels (Figs 1 and 5) and the variable exposure to nutrients and gases between the upper and lower layers. Therefore, a horizontal methodology was investigated as a possible alternative. Initial experimentation with this orientation included complex shapes and 3D computer-aided design for 3D printing of specific chambers (data not shown) but finally a simple, and inexpensive silicone half-well plug methodology was used with success (Fig. 2). In all four hydrogel types tested, the horizontal methodology to make a co-culture from two cell-encapsulated hydrogels appeared to work well at a gross level (Fig. 2) and when visualized using CLSM, both labelled cell types appeared to interact at a single interface as expected (Fig. 3). Despite these initial results, it was important to ascertain the cellular positions immediately after manufacture, to ensure that a single distinct interface was formed. In the horizontal arrangement, the side profile of the interface plane was assessed in all four hydrogel types (Fig. 5). Agarose, gellan and fibrin hydrogels displayed a clear, perpendicular interface region between the two cell populations (Fig. 5A–C), whereas collagen hydrogels resulted in an angled interface (Fig. 5D). The degree of angulation was caused by the integrity of the initial hydrogel and how well it maintained its shape when the silicone plug was removed from the well. Agarose and gellan hydrogels are more robust in nature at the concentrations used here, with a mechanical stiffness in the range of approximately 2 kPa for a 0.5% agarose gel and gellan hydrogels [19, 28] and so this is largely expected in these materials. Indeed, their roles in the food industry as thickening agents in food confirm their robust nature [29]. Conversely, collagen gels have much lower mechanical stiffnesses [30] and so this could explain the lack of mechanical integrity in collagen gels when the mould was removed. Surprisingly, despite fibrin gels apparent gross similarity to collagen gels, it worked very well in this system, and a perpendicular interface was achieved between the two cell-encapsulated hydrogels (Fig. 5D). In future experiments, it is clear that an important next step will be to evaluate the impact of different stiffnesses of hydrogel matrix for each cell type, as the surrounding matrix stiffness is known to affect cellular behaviour and ECM production *in vitro* in many different cell types [31–36].

Tracking cellular migration within the 3D *in vitro* model was also an important finding to characterize. Indeed, all four hydrogel types were subjected to the same area of gel visualization via CLSM on 4 consecutive time points, 24 h apart (Fig. 6). Although the cellular appearance in each gel was similar on Day 0, with interaction of the two cell populations at a single interface, distinct differences were observed between the cellular morphology between agarose and gellan and collagen and fibrin (Fig. 6). While fibrin and collagen appeared to support cellular attachment, with notable changes in cellular morphology as cells attached to the substrate (Fig. 6C and D), cells within gellan and agarose hydrogels remained rounded and unattached. Further differences were noted between collagen and fibrin too, where in fibrin hydrogels there was clear migration of tendon cells (green) into the bone cell region (red) of the hydrogels (Fig. 6C), migration through the collagen gels was not noticed during this time period (Fig. 6D). Overall, the fibrin hydrogel co-culture system appeared to display the most favourable characteristics for forming a co-culture model and so was the material taken forward into further experimentation.

A key finding from this study was the importance of the tissue culture plates used for creating the 3D *in vitro* models. Using standard tissue-cultured treated culture wells worked well for manufacturing the initial co-culture hydrogel model, however, visualization of the side-plan projections over time revealed a propensity for the cells to migrate towards the bottom of the well, therefore reducing the overall 3D nature of the co-culture (Fig. 7A). In order to counter this, non-tissue culture treated culture wells were used and retained the initial 3D arrangement of cells in the model (Fig. 7B). This is a notable finding and key for other researchers using 3D models to ensure that their well substrate does not provide a preferential attachment substrate for the duration of the experiment.

In addition to gaining an understanding of cellular location with the co-culture models, it was also vital to investigate the effect of the choice of hydrogel on cellular viability to ensure that cells remained viable throughout the experimental procedure. To this end, viability studies were performed on CTF and MC3T3 cells in both fibrin hydrogels as our experimental material and in agarose hydrogels, since our initial qualitative results appeared to show a lack of cellular attachment and movement within the substrates. Surprisingly, the percentage of viable CTF cells in agarose significantly increased in agarose hydrogels (Fig. 8A) but remained stable in the fibrin hydrogels. It was postulated that the heat at which the cells are encapsulated into the agarose gel solution (∼40°C) could have had a potential impact on the reduced cellular viability on Day 0; however, this was not seen in the MC3T3 cell population, indicating a potential sensitively to temperature in the CTF cell population. Importantly, as viability is expressed as a percentage, this does not necessarily indicate an increase in cell number, rather a change in the ratio of live: dead cells present. A potential reason for this could be the presence of enzymes in the foetal bovine serum present in supplemented growth medium that can act to digest dead cellular DNA and therefore alter the dead cell ratio found through the quantitative assessment [36, 37]. This was investigated over a period of 4 days with no significant effect (data not shown) but remains to be a potential factor that was influencing these cell populations. In MC3T3 cells, viability was unaffected between Days 0 and 7, but showed a significant reduction in viability when cultured in fibrin hydrogels at Day 7. The location of the dead cells appeared in the centre of the hydrogel indicating that nutrient transfer was potentially impacted within these samples. The live/dead assay relies on CLSM lasers penetrating through the various hydrogel materials in 3D, however the opacity of each hydrogel changes overtime. This non-controllable variable presented serious challenges to standardize data collection from the different material samples. In addition, fluorescent protein bleaching can affect the quality of the detected signal overtime (data not shown) and so for these reasons, another methodology to assess cell viability was needed.

Following inconclusive results from cell viability studies, a DNA-based cell proliferation assay was adopted which required cells to be retrieved from the hydrogels. The DNA content of agarose and fibrin hydrogels and each cell type was investigated to gain a better understanding of cellular growth within each material and cell combination (Fig. 9) and to form a better comparison between the qualitative images collected for each combination (seen in Fig. 8). DNA content increased in CTF cells cultured in agarose hydrogels (Fig. 9A) but conversely reduced in MC3T3 cells cultured in agarose hydrogels (Fig. 9B). Agarose has been used as a 3D scaffold before [36] and other studies have reported conflicting reports about cell viability in agarose. Ise et al. reported an increase in hepatocytes proliferation and viability after 21 days of culture when encapsulate in 3% agarose [38]. However, they have also reported decrease in viability and ultimately cell death when the same cell type was encapsulated in higher concentrations of agarose [38]. As the concentration of agarose used in the developed co-culture system was 1%, the high agarose concentration could have had the same effect on MC3T3 cells, but the same concentration of agarose did not affect the CTF cells. Another study has reported an increase in human osteosarcoma cell line (MG-63) cell density after 14 days of culture in a 1% agarose [39]. Overall, it seems likely that different cell types behave differently when encapsulated in agarose hydrogels.

The most striking result displayed was the effect of CTF and MC3T3 growth in fibrin gels, where DNA content was shown to significantly decrease over 7 days in culture (Fig. 9A and B). This result was most unexpected, largely due to the fact that fibrin is well known for being an excellent 3D scaffold material for various cell types [21–23, 40, 41] but also that the qualitative images presented in Figs 6 and 8 clearly demonstrated an increase in cellular number visualized via CLSM. From this, the methodology of cell retrieval from fibrin gels was revisited, as based on methodology present in Carrion *et al.* [27]. Encouragingly, the used of the enzyme nattokinase, facilitated appropriate scaffold digestions and release of cells to measure DNA content accurately (Fig. 9C). Here, we are now confident that fibrin hydrogels support cell growth in both CTF (Fig. 9Ci) and MC3T3 cells (Fig. 9Cii) that confirm its use in a co-culture for *in vitro* model development. As an important next step, the use of this co-culture model with primary cells from the same species is vital to confirm its usefulness as a 3D co-culture model for modelling a musculoskeletal interface *in vitro*.

### Conclusion

This study has investigated the different orientation, material and culture conditions that are required to build a reproducible *in vitro* 3D co-culture model. We propose that by using a simple and inexpensive silicone moulding procedure and cell-encapsulated fibrin gel hydrogels, a horizontally orientated and representative model of a musculoskeletal interface can be made. Future work will now investigate the use of this model to investigate the cellular and molecular factors involved in musculoskeletal tissue interface formation and as a model to direct and discover new therapeutic treatments for improving musculoskeletal injury and repair.

## Funding

This work was supported by the Tenovus Scotland E14/02. In addition, H.A. was supported via a Saudi Arabian Cultural Bureau scholarship.

*Conflict of interest statement.* None declared.
